# BMPRIA Mediated Signaling Is Essential for Temporomandibular Joint Development in Mice

**DOI:** 10.1371/journal.pone.0101000

**Published:** 2014-08-05

**Authors:** Shuping Gu, Weijie Wu, Chao Liu, Ling Yang, Cheng Sun, Wenduo Ye, Xihai Li, Jianquan Chen, Fanxin Long, YiPing Chen

**Affiliations:** 1 Department of Cell and Molecular Biology, Tulane University, New Orleans, Louisiana, United States of America; 2 Department of Dentistry, ZhongShan Hospital, FuDan University, Shanghai, P.R. China; 3 Guanghua School of Stomatology, Sun Yat-sen University, Guangzhou, Guangdong, P.R. China; 4 Academy of Integrative Medicine, Fujian University of Traditional Chinese Medicine, Fuzhou, Fujian, P.R. China; 5 Department of Internal Medicine, Washington University School of Medicine, St. Louis, Missouri, United States of America; University of Southern California, United States of America

## Abstract

The central importance of BMP signaling in the development and homeostasis of synovial joint of appendicular skeleton has been well documented, but its role in the development of temporomandibular joint (TMJ), also classified as a synovial joint, remains completely unknown. In this study, we investigated the function of BMPRIA mediated signaling in TMJ development in mice by transgenic loss-of- and gain-of-function approaches. We found that BMPRIA is expressed in the cranial neural crest (CNC)-derived developing condyle and glenoid fossa, major components of TMJ, as well as the interzone mesenchymal cells. *Wnt1-Cre* mediated tissue specific inactivation of *BmprIa* in CNC lineage led to defective TMJ development, including failure of articular disc separation from a hypoplastic condyle, persistence of interzone cells, and failed formation of a functional fibrocartilage layer on the articular surface of the glenoid fossa and condyle, which could be at least partially attributed to the down-regulation of *Ihh* in the developing condyle and inhibition of apoptosis in the interzone. On the other hand, augmented BMPRIA signaling by *Wnt1-Cre* driven expression of a constitutively active form of *BmprIa* (*caBmprIa*) inhibited osteogenesis of the glenoid fossa and converted the condylar primordium from secondary cartilage to primary cartilage associated with ectopic activation of Smad-dependent pathway but inhibition of JNK pathway, leading to TMJ agenesis. Our results present unambiguous evidence for an essential role of finely tuned BMPRIA mediated signaling in TMJ development.

## Introduction

As an evolutionary creature, the temporomandibular joint (TMJ) is a unique synovial joint generated only in mammals and is involved in food capture and intake, speech, as well as maturation of the facial contour [1]. It is made of specific components originated from the skull base and the low jaw including the glenoid fossa, condyle, articular disc, ligaments, and joint capsule. Although defined as a synovial joint, the developmental process of TMJ differs significantly from the joints of appendicular skeletons that are generated by cleavage or segmentation within a single skeletal condensation [2]. The TMJ develops from two distinct mesenchymal condensations, the glenoid fossa blastema that ossifies primarily through intramembranous bone formation, and the condylar blastema that undergoes endochondral ossification. These two primordia are initially separated widely by intervening mesenchyme that was thought to later contribute to the articular disc and capsule, as well as the synovial lining of joint cavity [3], [4] Subsequently, the condylar primordium, arising from the periosteum of the mandibular bone and therefore classified as secondary cartilage [5], [6], grows rapidly towards the glenoid fossa, and meanwhile, the articular disc forming from a condensed stripe flanking the apex of the condyle and subsequently separating from the latter, divides the interzone into the upper and lower joint cavities [7]. In mice, the mesenchymal condensation of condyle appears at embryonic day 13.5 (E13.5) and the glenoid fossa at E14.5 [8]. At E15.5, the shape of glenoid fossa and condyle has been established, and at E16.5, the upper synovial cavity becomes discernible with a disc beginning to form. Subsequently at E17.5, the lower joint cavity appears as a definite articular disc separates from the apex of the condyle. This intricate multi-step developmental process is regulated by intrinsic and extrinsic factors.

As for intrinsic constituents, the significance of genetic factors has attracted the attention of the field. Gene targeting studies have revealed essential roles for a number of transcription factors and growth factors in TMJ development, as evidenced by the absence of condylar cartilage in mice carrying mutations in *Sox9*, *Runx2*, or *Tgfbr2*, and by the abnormal development of mice carrying mutations in *Shox2* or *Spry1* and *Spry2* [8]–[14]. Ihh, which plays a pivotal role in long bone development and digit joint formation [15], has been implicated in TMJ development by initiating the formation of articular disc and instructing the disc to undergo proper morphogenesis and to separate from the condyle, as well as in maintaining proper structure and function of the TMJ after it forms [16]–[18]. Lack of Ihh or its downstream effector Gli2 results in missing of a distinct disc in the TMJ [16], [17]. In addition, extrinsic factors such as biomechanical force also contribute to TMJ development [2].

Bone morphogenetic proteins (BMPs) exert diverse biological functions during development and postnatal homeostasis. BMP signals are transduced into cells through the type I and type II transmembrane serine/threonine kinase complexes by activating Smad-dependent (canonical) pathway, as well as Smad-independent (non-canonical) pathway via activation of the mitogen-activated protein kinase (MAPK) signaling [19]. Extensive studies have established critical roles for BMP signaling in skeletal development and joint morphogenesis, particularly in joint formation of long bones. Joint formation in the appendage skeletons begins with the formation of a condensed cell stripe known as interzone in the developing cartilage template [20]. Cells in the edges of the interzone give rise to the articular cartilage that covers the ends of the adjacent skeletal elements, while cells in the middle of the interzone undergo programmed cell death, leading to physical separation of the contiguous cartilage element and formation of joint cavity [21]. Several members of BMP family, including *Bmp2*, *Bmp4*, *Gdf5*, *Gdf6*, *Gdf7*, are expressed in the interzone along with BMP antagonists *Chondin* and *Noggin* [22]–[26]. Mice carrying mutations in *Gdf5* or *Gdf6* exhibit lack of joint formation at specific locations [25], [27], demonstrating a direct action of BMP signaling in joint morphogenesis. On the other hand, elevated BMP signaling also blocks joint formation, as manifested by failure in joint formation in the limbs of *Noggin* mutant mice [28]. These loss-of- and gain-of-function studies indicate an essential role for tightly regulated BMP activity in synovial joint formation. Furthermore, BMP signaling is also involved in postnatal joint homeostasis and tissue remodeling [26], [29].

Being one of the two primary BMP type I receptors (BMPRIA and BMPRIB), BMPRIA plays crucial roles in skeleton patterning and development. In developing limb skeletons, *BmprIa* is expressed in the joint interzone, perichondrium, periarticular cartilage, and hypertrophic chondrocytes [26], [30]–[32]. Tissue specific deletion of *BmprIa* in cartilage lineage leads to chondrodysplasia attributed at least partially to the defective cell proliferation as well as the premature hypertrophy of chondrocytes associated with down-regulation of *Ihh* [33], [34]. While joint defect was not identified in mice carrying cartilage specific inactivation of *BmprIa*, mice carrying tissue specific inactivation of *BmprIa* in the interzone indeed exhibited missed joints in the ankles [26], indicating a requirement of BMPRIA mediated signaling in joint formation.

Despite a wealth of documents on BMP signaling in bone and joint formation of appendicular skeletons, little is known about its role in TMJ development. Thus far, the only line of evidence implicating a possible involvement of BMP signaling in TMJ formation is that *Bmp2* and *Bmp7* were found to be expressed in the developing condyle [17], [35]. To gain an insight into BMP signaling in TMJ development, in this study, we used transgenic loss-of- and gain-of-function approaches to investigate the function of BMPRIA mediated signaling in TMJ development.

## Materials and Methods

### Ethics statement

Experiments that involved use of animals in this study was approved by the Institutional Animal Care and Use Committee (IACUC) of Tulane University (protocol number: 0367R) and was in strict accordance with the recommendations in the Guide for Care and Use of Laboratory Animals of the National Institutes of Health.

### Animal and sample collection

The generation and identification of transgenic and gene-targeted animals, including *Wnt1-Cre, BmprIa^f/f^, and pMes-caBmprIa* that carries a conditional constitutively active form (with Gln203 to Asp change) of *BmprIa* transgenic allele, have been described previously [15], [31], [36], [37]. *Wnt1-Cre;BmprI ^f/f^* embryos were obtained by crossing *Wnt1-Cre;BmprIa^f/+^* mice with *BmprIa^f/f^* line. *Wnt1-Cre;pMes-caBmprIa* embryos were generated by mating *Wnt1-Cre* mice with *pMes-caBmprIa* transgenic line. *Ihh* null mutants were harvested from intercross of *Ihh* heterozygous mice. Embryos with *BmprIa* deficiency in their neural crest cells (*Wnt1-Cre;BmprIa^f/f^*) die around E12.5 due to norepinephrine depletion [38], [39]. To prevent early embryonic lethality, pregnant females were administrated with the β-adrenergic receptor agonist from 7.5 postcoitum (dpc) on by supplementing drinking water of dams with 200 µg/ml isoproterenol, which would allow *Wnt1-Cre;BmprIa^f/f^* embryos to survive to term [40], [41]. Embryos were collected from the timed pregnant females, and head samples were dissected in ice cold PBS, fixed individually in 4% paraformaldehyde (PFA) or z-fix (ANATECH Ltd; #170) overnight at 4°C, and tail clip from each embryo was used for PCR-based genotyping, respectively. Mutant and control heads were positioned for serial coronal sections through the TMJ. Comparable sections through the apex of the condyle were picked up for histological, in situ hybridization, immunostaining, and cell apoptosis analyses.

### Histology, in situ hybridization, and immunohistochemistry, and Tunel assay

For histological study, paraffin sections were made at 6 µm and subjected for standard Hematoxylin/Eosin staining or Azoncarmine G/Aniline blue staining, as described [42]. Five mutant samples at each stage examined were used to ensure consistency of the phenotype. For in situ hybridization analyses, sections were cut at 10 µm and pretreated with proteinase K and hybridized with appropriate probes. Transcripts were detected by color reaction using BM purple (Roche) as described previously [43]. For immunohistochemical staining, frozen sections, made at 8 µm, were blocked with 4% goat serum and then incubated with primary antibodies against BMPRIA (Abcam; ab38560), Lubricin (Santa Cruz; sc-9854), pSmad1/5, pJNK, pERK, and p-p38 (from Cell Signaling; #9516, #9255, #4370, and #9211), respectively, at 4°C overnight. After washing, samples were incubated with secondary antibodies (Alexa Fluor488 goat anti-rabbit IgG from Invitrogen; #A-11034), counterstained with DAPI, and visualized under fluorescent microscope. Negative controls without primary antibodies were included in parallel. At least three samples of each genotype were used for histology, in situ hybridization, and immunohistochemistry analyses. Terminal deoxynucleotidyl transferase dUTP nick end labeling (Tunel) assay was applied to detect apoptotic cells using In Situ Cell Death Detection Kit (Roche), as described previously [44], [45]. Three samples of each genotype were subjected to Tunel assays. Tunel-positive cells within the interzone were counted and presented as percentage of total cells within arbitrarily defined areas. Student's *t*-test was used to determine the significance of difference between wild type controls and mutants, and the results were presented as *P* value.

## Results

### Inactivation of *BmprIa* in CNC lineage leads to defective TMJ formation

**Figure 1:**
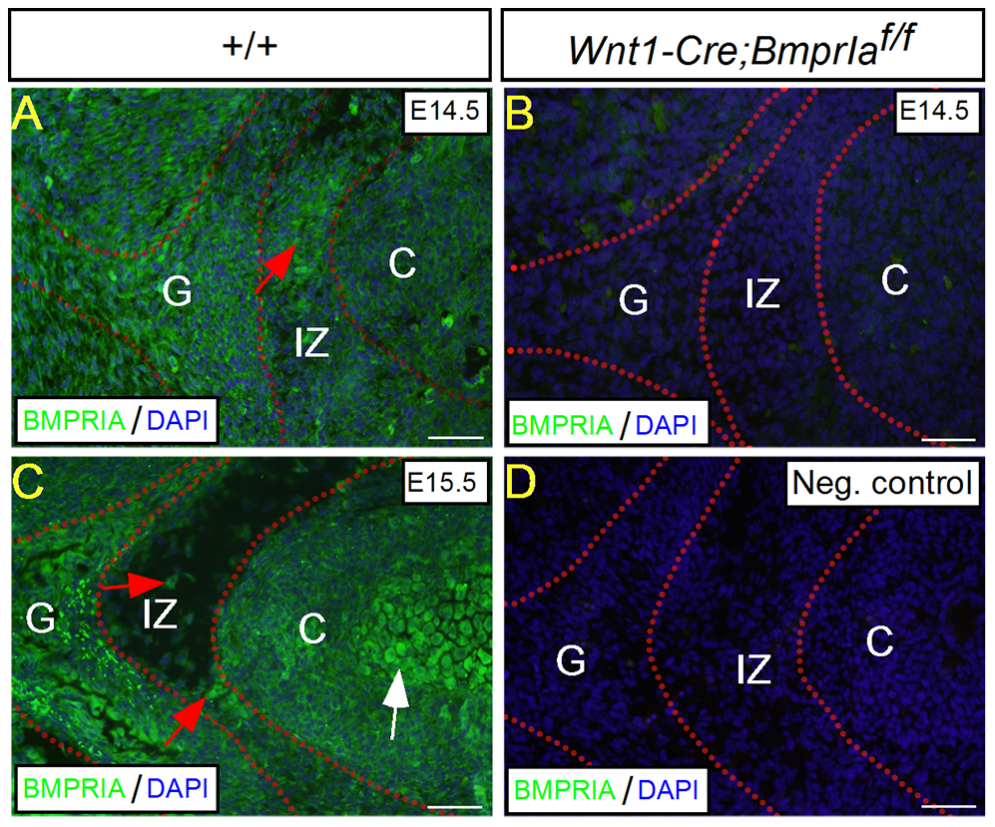
Expression of BMPRIA in the developing TMJ. (A–D) Immunohistochemistry shows expression of BMPRIA in the condylar cartilage, interzone, and glenoid fossa of E14.5 (A) and E15.5 (C) wild type animals, but a lack of staining on the *Wnt1-Cre;BmprIa^f/f^* TMJ (B) and on the negative control (*D*). Red arrows point to positive staining in the interzone, and white arrow points to the hypertrophic region where strong expression is detected. Abbreviation: C, condyle; G, glenoid fossa; IZ, interzone. Scale bar = 100 µm.

To investigate the role of *BmprIa* in TMJ development, we began with examination of BMPRIA expression by immunohistochemistry. At E14.5 when both the primordial condyle and glenoid fossa become discernible, BMPRIA was found present in the developing condyle and glenoid fossa as well as the interzone, with a relatively low level in the condylar cartilage (Fig. 1A). At E15.5, BMPRIA expression retained in the condyle, glenoid fossa, and interzone, with an increased level in chondrocytes undergoing hypertrophy (Fig. 1C). This expression pattern is similar to that in the developing limb skeleton including the joints [26], [30]–[32], suggesting a role for BMPRIA in TMJ morphogenesis. Since the condyle, glenoid fossa, and interzone cells are all derived from CNCs [8], we inactivated *BmprIa* in CNC lineage using the *Wnt1-Cre* transgenic allele. Immunohistochemistry confirmed the absence of BMPRIA in the developing TMJ of *Wnt1-Cre;BmprIa^f/f^* mice (Fig. 1B).

**Figure 2:**
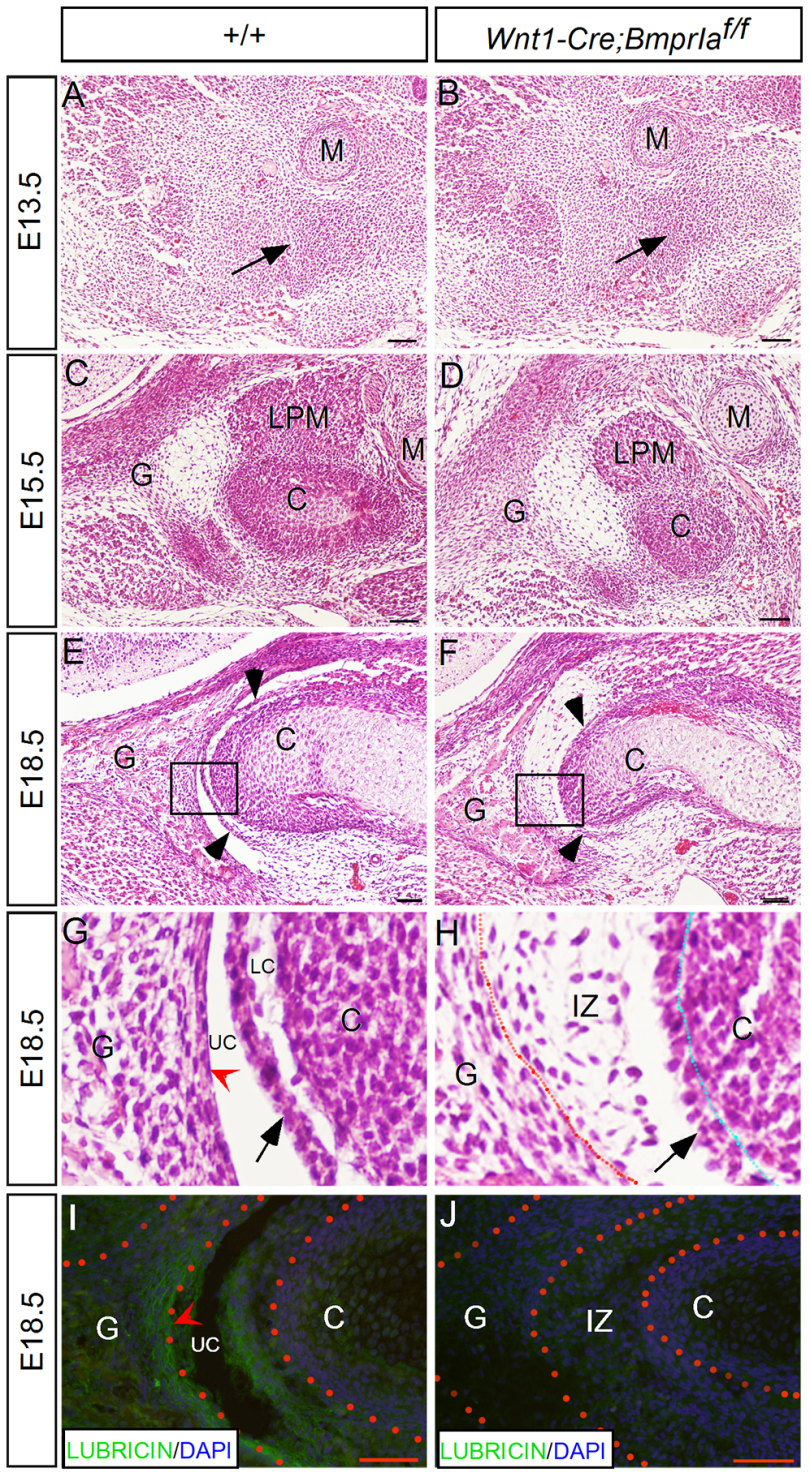
*Wnt1-Cre;BmprIa^f/f^* mice display TMJ defects. (A–H) H&E staining shows histology of the developing TMJ of wild type controls (A, C, E, G) and mutants (B, D, F, H). Note that the initial condensation of the condylar anlagen at E13.5 (A, B) and the morphology of the glenoid fossa at E15.5 (C, D) appear comparable between the controls and mutants. However, the size of the mutant condyle is reduced at E15.5 (D). At E18.5, distinct structures including a definite disc, the upper and lower joint cavities, and the articular surface of the glenod fossa are well present in the control TMJ (E, G). However, in mutants, while a disc-like compact layer could be identified closely associated with the apex of the condyle, it fails to separate to form a distinct disc. In addition, the interzone cells persist, and a fibrocartilage layer fails to form on the articular surface of the glenoid fossa (F, H). (I, J) Immunnohistochemistry reveals expression of Lubricin in the synovial membrane of the control TMJ (I), and the complete absence of Lubricin in the mutant TMJ (J). Arrows in (A, B) point to the condylar condensation, and in (G, H) point to the disc. Arrowheads in (E, F) point to the disc. Red arrowhead points to the articular surface in (G) and the synovial membrane in (I). Abbreviation: C, condyle; G, glenoid fossa; M, Meckel's cartilage; IZ, interzone; LC, lower cavity; UC, upper cavity; LPM, lateral pterygoid muscle. Scale bar = 50 µm.

Histological analyses showed that the initial condensation of the condylar anlage in mutant mice appeared comparable to littermate controls at E13.5 (Fig. 2A, 2B). At E15.5, the morphology of the glenoid fossa did not exhibit an obvious difference between mutants and controls, but the size of the mutant condyle was reduced as compared to controls (Fig. 2C, 2D). At E18.5, the control TMJ displayed distinct structures, including a definite articular disc, the upper and lower synovial cavities, and the fibrocartilage/synovial membrane on the articular surface of the glenoid fossa and condyle (Fig. 2E, 2G). However, at this stage, the mutant TMJ exhibited a number of severe defects, including a hypoplastic condyle, lack of a definite disc, failed formation of an upper joint cavity evidenced by the existence of loose connective tissue in the interzone, as well as the absence of the fibrocartilage/synovial membrane of the glenoid fossa (Fig. 2F). Close examination of the mutant condyle at E18.5 revealed the formation of a disc-like structure that failed to separate from the apex of the condyle, leading to an absence of the lower joint cavity (Fig. 2F, 2H). The lack of a synovial joint cavity in the *Wnt1-Cre*;*BmprIa^f/f^* TMJ was further confirmed by the absent expression of Lubricin, a key component of joint fluids [46], [47], as compared to its abundant expression in controls (Fig. 2I, 2J).

### Delayed chondrocyte maturation in the *Wnt1-Cre*;*BmprIa^f/f^* condyle

**Figure 3:**
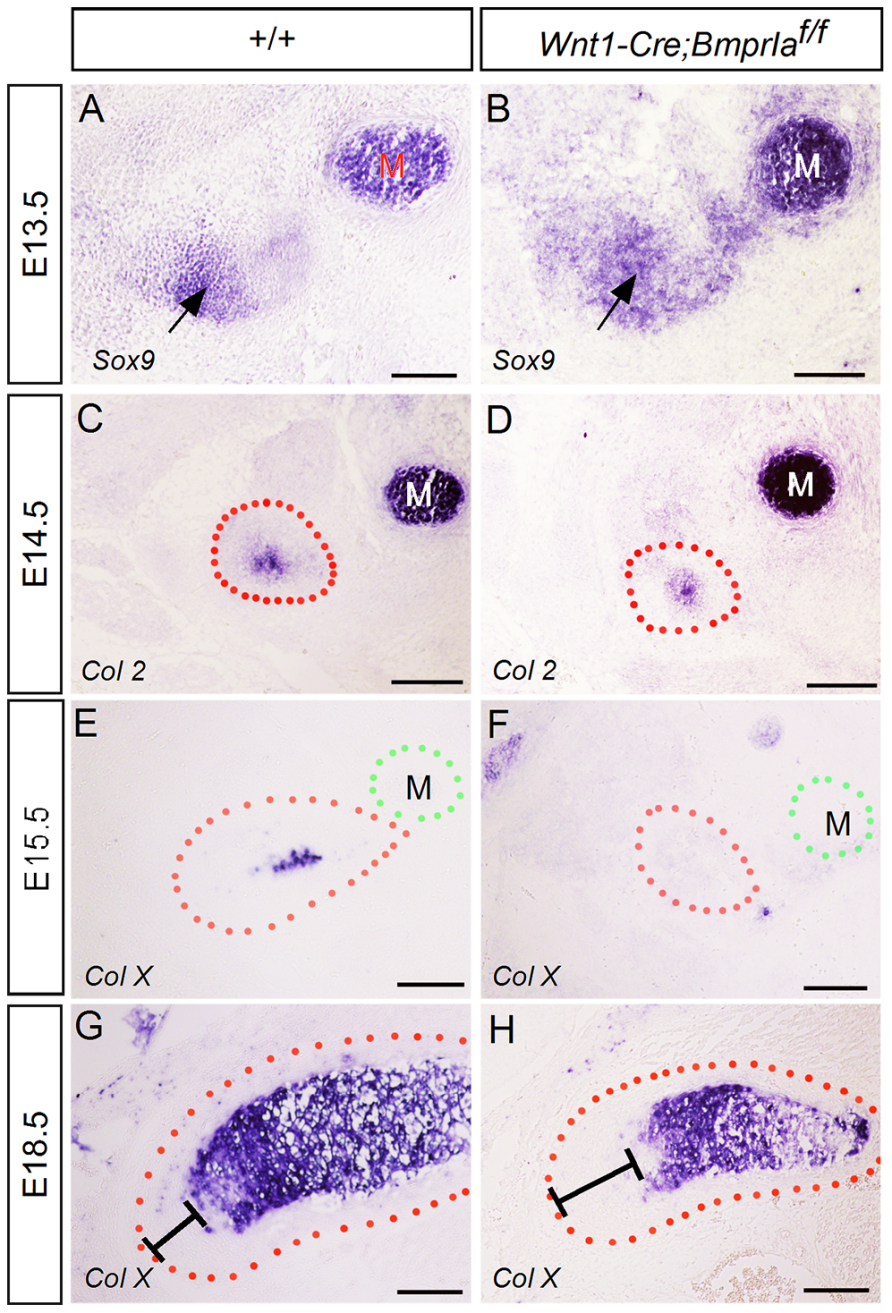
Delayed hypertrophic differentiation in the *Wnt1-Cre;BmprIa^f/f^* condylar cartilage. (A, B) In situ hybridization shows *Sox9* expression in the condylar condensation (arrow) of wild type (A) and mutant (B) at E13.5. (C, D) *Col II* expression exhibits comparable pattern in the condyle of wild type (C) and mutant (D) at E14.5. (E–H) In situ hybridization reveals *Col X* expression in the wild type condyle at E15.5 (E) and E18.5 (G). However, *Col X* expression is not detected in the mutant condyle at E15.5 (F), but is seen at E18.5 (H). The distance between the apex of the condyle and the beginning of hypertrophic zone is longer than that in the control condyle (G, H). Abbreviation: M, Meckel's cartilage. Scale bar = 100 µm.

Despite being a secondary cartilage, the growth of condylar cartilage takes the similar chondrogenesis and endochondral ossification process as that in long bone formation. Since BMPRIA mediated signaling is known to regulate primary cartilage differentiation [33], [34], we set to examine chondrogenic differentiation process in the *Wnt1-Cre;BmprIa^f/f^* condyle. In the developing condyle, mesenchymal condensation appears at E13.5, and chondrogenic differentiation occurs at E14.5, and hypertrophy initiates at E15.5 [8]. We found that the timing of initial condensation of the condylar anlagen, as indicated by the expression of *Sox9*, and chondrogenic differentiation, determined by *Col II* expression, was comparable between wild type controls and *Wnt1-Cre*;*BmprIa^f/f^* mice (Fig. 3A–D). However, the mutant condyle exhibited a delayed terminal hypertrophy of chondrocytes, as assessed by the delayed *Col X* expression (Fig. 3E–H), and the longer distance between the apex and the beginning of hypertrophic zone in the mutant condyle at E18.5 (Fig. 3G, 3H). This phenotype differs from that in long bones where inactivation of *BmprIa* in condrocytes causes premature chondrocyte differentiation [34], likely due to different properties of primary v.s secondary cartilage.

### Down-regulation of *Ihh* and inhibition of apoptosis in the *Wnt1-Cre*;*BmprIa^f/f^* TMJ

**Figure 4:**
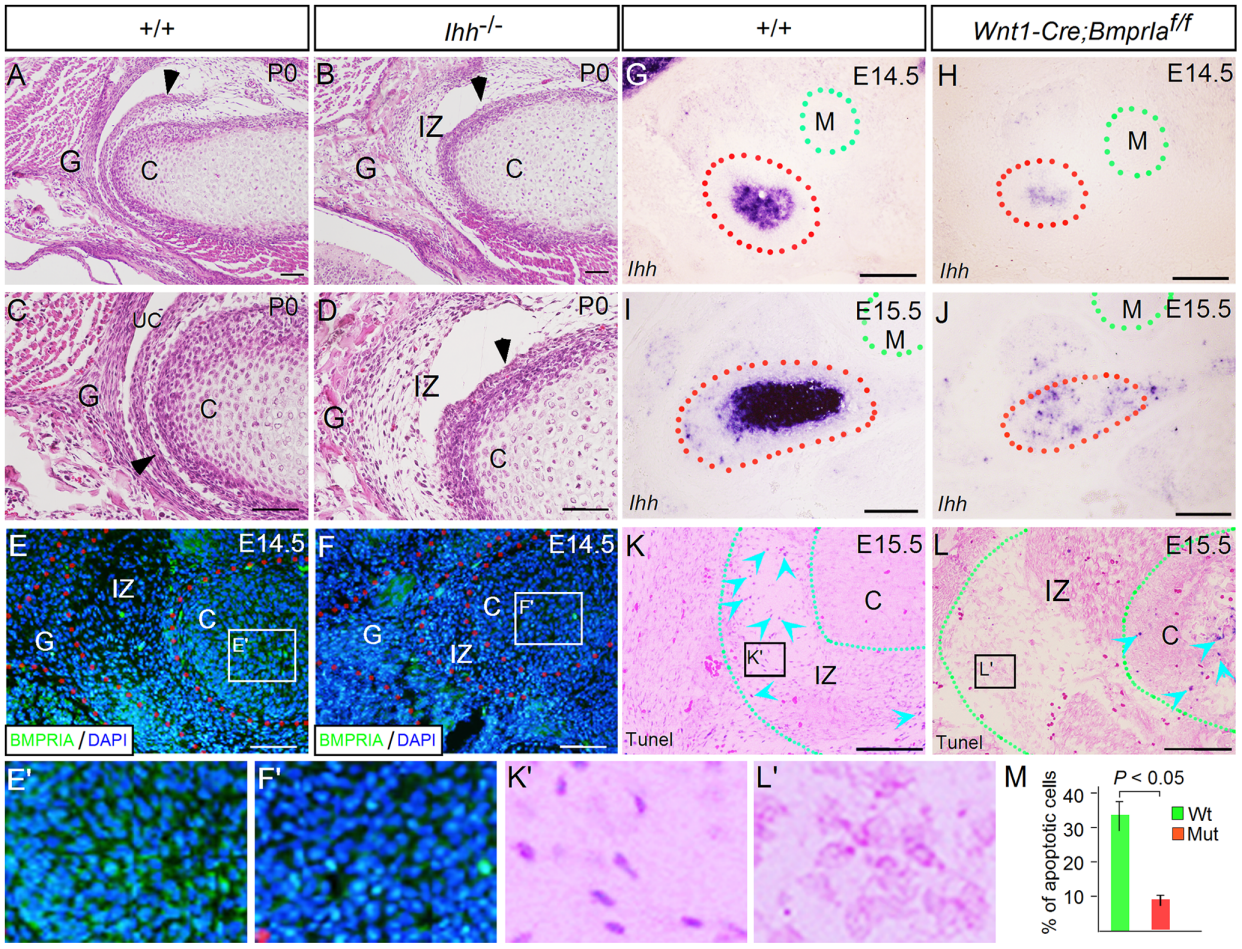
TMJ defects in *Ihh* mutants and reduced *Ihh* expression in the *Wnt1-Cre;BmprIa^f/f^* condyle. (A–D) H&E staining reveals TMJ defects in the *Ihh* mutant TMJ (B, D), as compared to control (A, C) at P0. In mutant, a disc-like structure (arrowhead) forms but fails to separate from the apex of the condyle, the fibrocartilage layer fails to form on the articular surface of the glenoid fossa, and the interzone cells persist (B, D), as compared to the formation of distinct TMJ structures, including the disc (arrowhead), the fibrocartilginous articular surface, and the clear upper joint cavity, in controls (A, C). (E, E′, F, F′) Immunohistochemistry reveals significantly down-regulated BMPRIA expression in the condylar cartilage and interzone of *Ihh* mutant at E14.5 (F, F′), as compared to littermate control (E, E′). (G–J) In situ hybridization shows a dramatic down-regulation of *Ihh* in the condylar cartilage of *Wnt1-Cre;BmprIa^f/f^* mice at both E14.5 (H) and E15.5 (J), as compared to controls (G, I). Tunel assay reveals numerous apoptotic cells (arrowheads) in the interzone of wild type control at E15.5 (K, K′), but very few apoptotic cells in the interzone of the *Wnt1-Cre;BmprIa^f/f^*TMJ at the same age (L, L′). In contrast, some apoptotic cells (arrowheads) were observed in the mutant condyle (L). (M) Comparison of the percentage of apoptotic cells in the interzone of controls and mutants. Standard deviation values were indicated as the error bars, and the Student's *t*-test was used to determine the significance of difference between control and mutant, as presented as *P* value. Abbreviation: C, condyle; G, glenoid fossa; M, Meckel's cartilage; IZ, interzone; UC, upper cavity. Scale bar = 100 µm.

The similar TMJ phenotype between *Wnt1-Cre;BmprIa^f/f^* mice and the mice carrying mutations in *Ihh* or its downstream effectors [16], [17], particularly the failure of disc separation, persistent interzone cells, and lack of fibrocartilaginous articular surface layer of the glenoid fossa in both mutants (Fig. 2F, 2H; Fig. 4B, 4D), and the overlapped expression pattern of *BmprIa* with *Ihh* in the developing condyle (Fig. 1) [8], [17], prompted us to examine *Ihh* expression in the developing condyle of *Wnt1-Cre;BmprIa^f/f^* mice. In situ hybridization assay revealed a dramatic down-regulation of *Ihh* expression in the developing condyle of the mutants at E14.5 and E15.5, as compared to controls (Fig. 4G–J), consistent with the role of BMPRIA as a positive regulator of *Ihh* expression [34], [48]. *BmprIa* thus likely acts through *Ihh* to regulate TMJ formation.

Because *BmprIa* is also expressed in the interzone mesenchymal cells that contribute to the articular disc and synovial membrane of the TMJ, the lack of fibrocartilage layer of the glenoid fossa and the persistence of interzone cells in the *Wnt1-Cre;BmprIa^f/f^* TMJ could be attributed either directly to the lack of *BmprIa* in these cells or indirectly to the significantly reduced level of *Ihh* in the condyle. To distinguish these alternatives, we conducted immunohistochemistry to examine BMPRIA expression in the *Ihh* ^-/-^ TMJ at E14.5. We found that although BMPRIA expression appeared comparable in the glenoid fossa of controls and mutants, its expression level was significantly reduced in the interzone and the condyle of the *Ihh* mutant TMJ (Fig. 4E–4F′).

While the mechanism of cavitation in TMJ development remains to be addressed, programmed cell death in the interzone is regarded as a critical cellular mechanism for joint cavity formation in long bones [21]. Since BMPRIA mediated signaling is required for programmed cell death in the limb, particularly in the interdigital region [26], [49], we wondered if the persistence of the loose connective tissue in the interzone of the *Wnt1-Cre;BmprIa^f/f^* TMJ is a consequence of reduced level of apoptosis. Tunel assay indeed revealed abundant apoptotic cells specifically in the interzone of the control TMJ at E15.5 (Fig. 4K, 4K′). In contrast, Tunel assay detected a significantly reduced level of apoptotic cells in the interzone as well as some apoptotic cells in the condyle of the mutant TMJ (Fig. 4L, 4L′, 4M). These observations suggest that similar to joint cavity formation in long bones, programmed cell death in the interzone also represents a critical cellular mechanism for joint cavitation during TMJ formation.

### Augmented BMPRIA signaling in CNC lineage leads to TMJ agenesis

**Figure 5:**
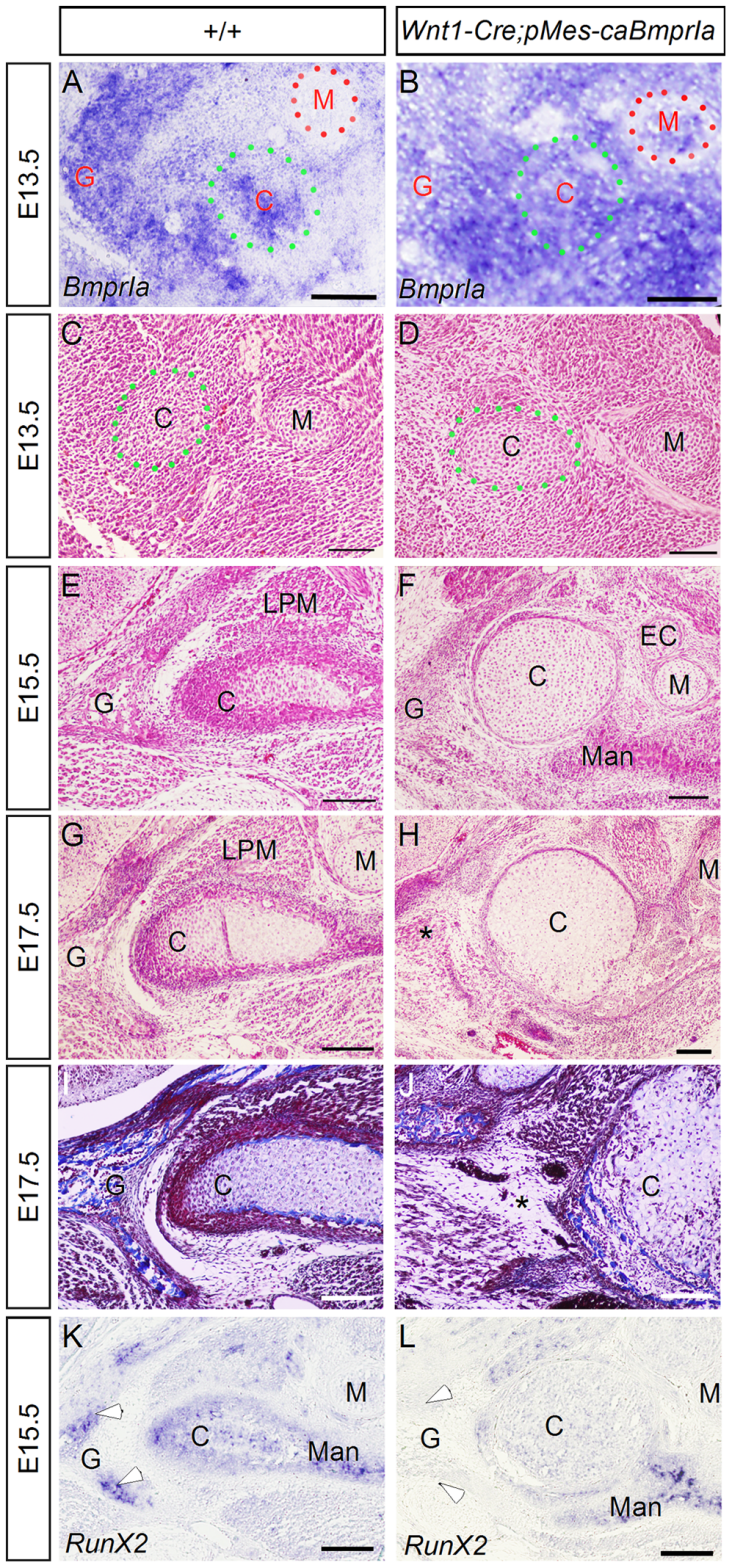
Augmented BMPRIA signaling in CNC cells leads to TMJ agenesis. (A, B) In situ hybridization shows expression of *BmprIa* in the condylar condensation, the future glenoid fossa forming site, and the interzone region, but not in Meckel's cartilage of an E13.5 wild type embryo (A), and an enhanced *BmprIa* expression in the TMJ forming site as well as the surrounding tissues including Meckel's cartilage in an E13.5 *Wnt1-Cre;pMes-cBmprIa* embryo (B). (C–H) H&E staining reveals initial condensation of condylar anlagen in control and transgenic animals at E13.5 (C, D), growth and differentiation into primary cartilage of the condylar cartilage and lack of osteogenesis in the glenoid fossa in the transgenic TMJ at E15.5 (F) and E17.5 (H). (I, J) Azocarmine G/Aniline blue staining reveals glenoid fossa degeneration, evidenced by lack of bone formation, in transgenic mouse (J), as compared to control (I). (K, L) In situ hybridization assay shows expression of *Runx2* in the forming gelnoid fossa, perichondral region of the condyle, and mandibular bone of an E15.5 wild type control (K), but an absent expression of *Runx2* in the glenoid fossa and a reduced expression in the condylar cartilage of an E15.5 transgenic animal (L). Note retention of *Runx2* expression in the mandibular bone of transgenic mouse (L). Asterisk in (H, J) indicates the site of glenoid fossa degeneration. Open arrowheads in (K, L) point to *Runx2* expression sites in the glenoid fossa. Abbreviation: C, condylar cartilage; G, glenoid fossa; M, Meckel's cartilage; EC, ectopic cartilage; LMP, lateral pterygoid muscle; Man, mandibular bone. Scale bar = 100 µm.

To further investigate the role of BMPRIA signaling in TMJ morphogenesis, we took a gain-of-function approach by transgenic expression of a constitutively active form of *BmprIa* (*pMes-caBmprIa*) [37] in CNC cells using the *Wnt1-Cre* allele. In situ hybridization revealed expression of *BmprIa* in the condensing condylar blastema, the forming site of glenoid fossa, and cells between them, but not in Meckel's cartilage of wild type embryo at E13.5 (Fig. 5A). In *Wnt1-Cre;pMes-caBmprIa* mice at the same stage, strong and wide spread expression of *BmprIa* was found in the TMJ forming region and its surrounding tissues including Meckel's cartilage, indicating successful transgenic expression of *BmprIa* in CNC lineage (Fig. 5B). We and others have shown previously that elevated BMPRIA mediated signaling in CNC cells leads to a spectrum of craniofacial bone defects, including cleft secondary palate, ectopic cartilage formation, and craniosynostosis [50], [51]. Histological analysis of the developing TMJ of *Wnt1-Cre;pMes-caBmprIa* mice identified unique TMJ developmental defects. Although the condensation of the condylar blastema occurred similarly to controls at E13.5 (Fig. 5C, 5D), the size of the condylar cartilage in transgenic animals became noticeably enlarged and the entire condylar cartilage appeared to become hypertrophic at E15.5, as compared to controls (Fig. 5E, 5F). Furthermore, unlike in controls that osteogenesis has begun in the glenoid fossa at this stage, the transgenic glenoid fossa failed to take osteogenic differentiation. At E17.5, the glenoid fossa became degenerated in transgenic animal (Fig. 5H). The failure of osteogenic differentiation in the glenoid fossa was further confirmed by the lack of bone formation in the glenoid fossa, assessed by Azocarmine G/Aniline blue staining, and by the absent expression of *Runx2*, a molecular marker for osteoblasts (Fig. 5I–L). Additionally, despite its expression in the mandibular bone, *Runx2* expression is also down-regulated in the condylar cartilage of *Wnt1-Cre;pMes-caBmprIa* mice (Fig. 5L). By E18.5, both the condyle and glenoid fossa degenerated and became unrecognizable (data not shown).

### Enhanced BMPRIA signaling converts the condylar primordium from secondary cartilage to primary cartilage by ectopic activation of canonical signaling and inhibition of JNK signaling

**Figure 6:**
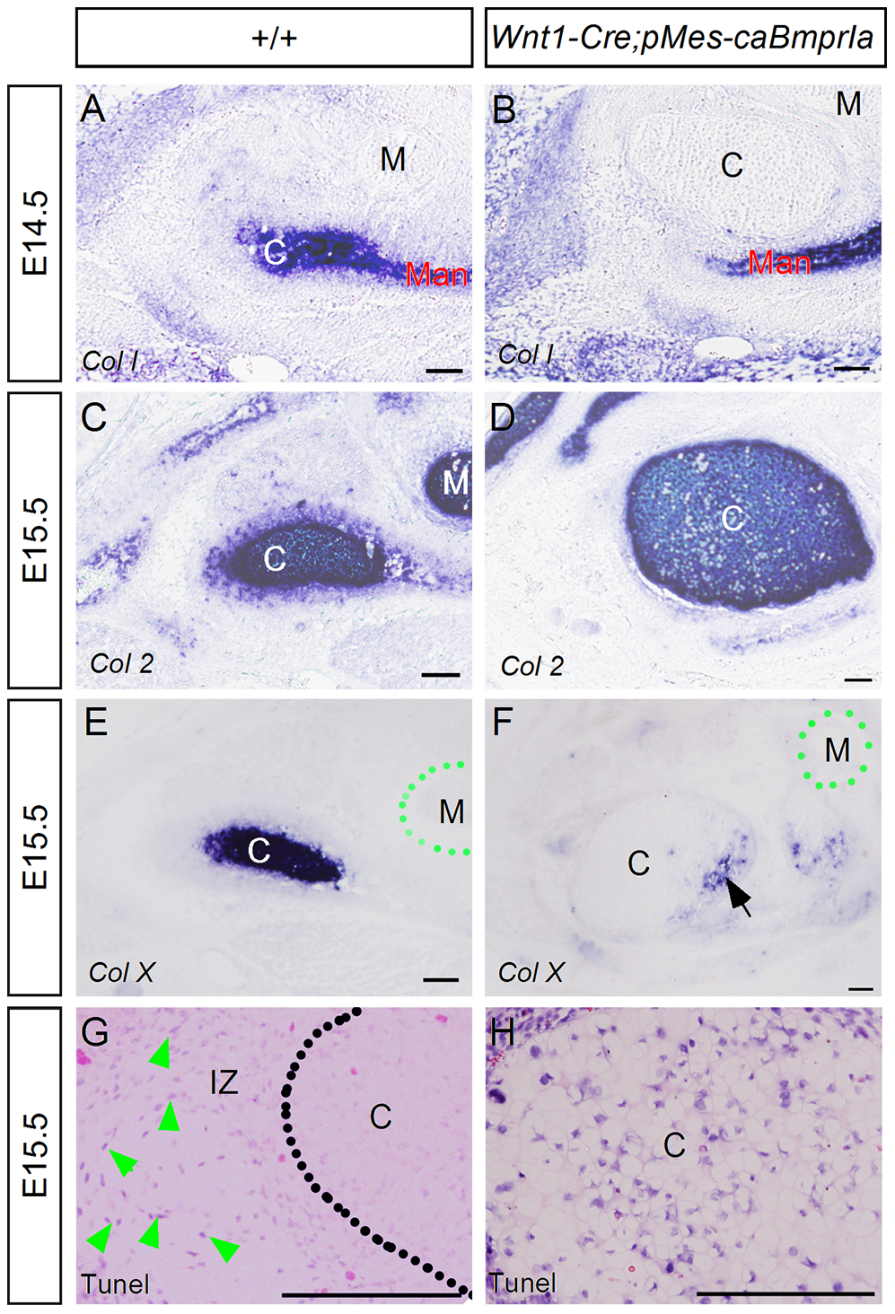
Elevated BMPRIA signaling converts secondary cartilage of the condylar primordium to primary cartilage and induces extensive cell death. (A–F) In situ hybridization detects *Col I* expression in the condylar cartilage and mandibular bone of an E14.5 control embryo (A), and in the mandibular bone of an E14.5 *Wnt1-Cre,pMes-caBmprIa* mice (B). However, *Col I* expression is not detected in the condylar cartilage of transgenic animal (B). *Col II* (C, D) and *Col X* (E, F) expression is observed in the condylar cartilage of control (C, E) and transgenic embryo (D, F) at E15.5. (G, H) Tunel assay reveals numerous apoptotic cells in the interzone but not in the condyle of the E15.5 wild type TMJ, but extensive cell death in the condylar cartilage of E15.5 transgenic embryo (H). Arrow in (F) points to *Col X* expression domain, and arrowheads in (G) point to apoptotic cells. Abbreviation: C, condylar cartilage; M, Meckel's cartilage; IZ, interzone. Scale bar = 100 µm.

As a secondary cartilage, the condylar cartilage expresses type I collagen (Col I), making it distinct from the primary cartilage [52]. Because of its aberrant differentiation, we wondered if the condylar cartilaginous element of *Wnt1-Cre;pMes-caBmprIa* mice retained its secondary cartilage characteristics. In situ hybridization assay revealed *Col I* expression in the control condylar cartilage and mandibular bone, but the absence of *Col I* in the transgenic condylar cartilaginous element despite its expression in the mandibular bone at E14.5 (Fig. 6A, 6B). However, the expression of *Col II* and *Col X* in the transgenic condylar cartilage confirmed its cartilage fate (Fig. 6C–F). We thus conclude that the *Wnt1-Cre;pMes-caBmprIa* condylar primordium adopts a fate of primary cartilage in response to an augmented BMPRIA-mediated signaling. Moreover, Tunel assay revealed an extensive apoptotic event in the transgenic condylar cartilage, beginning at E15.5, as compared to the lack of apoptosis in the control condyle at the same stage (Fig. 6G, 6H), which apparently contributes to the degeneration and disappearance of the condylar cartilage in transgenic animals.

**Figure 7:**
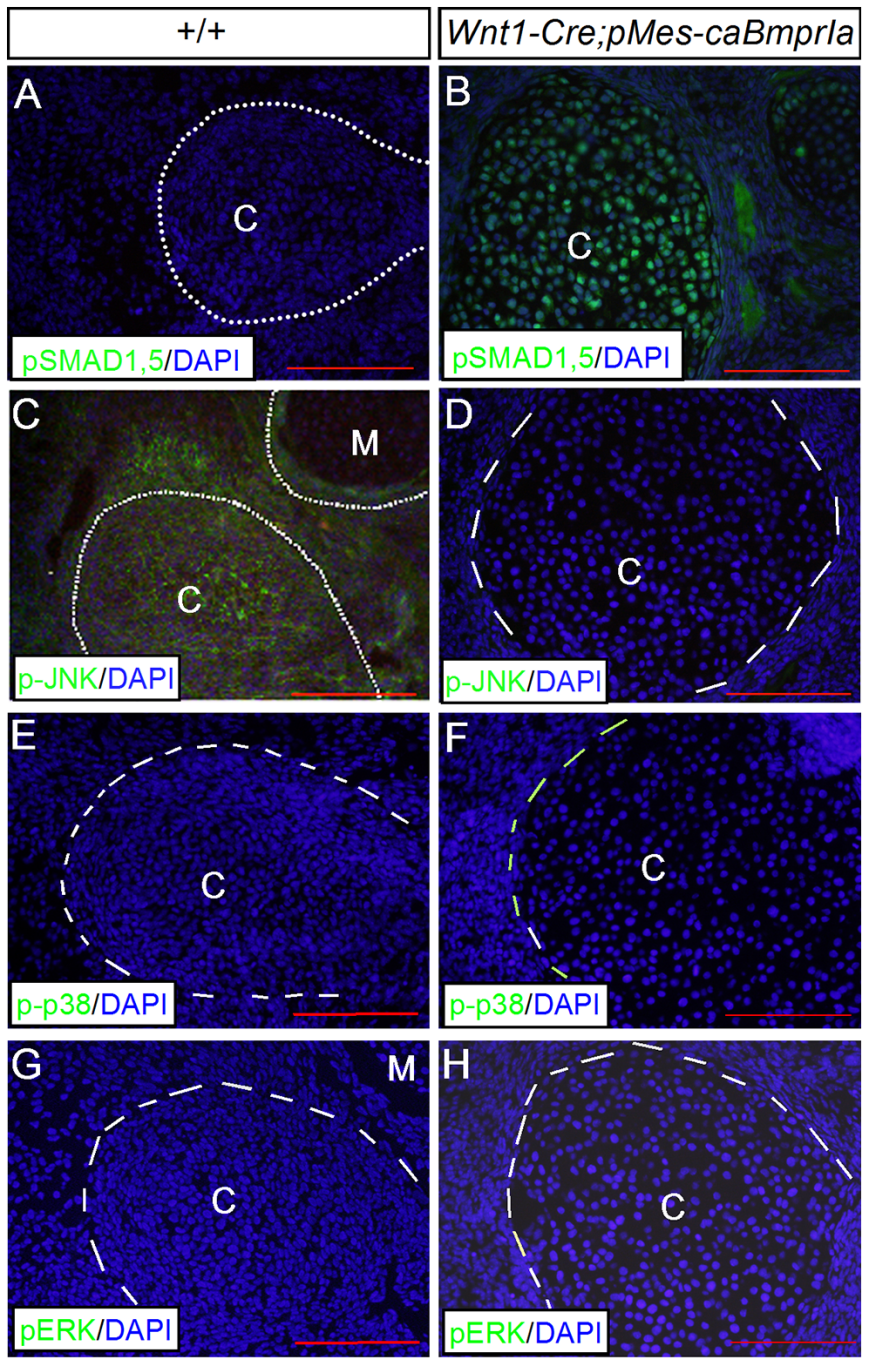
Enhanced BMPRIA signaling activates Smad-dependent pathway but inhibits JNK signaling pathway in the condylar cartilage. Immunohistochemistry shows absent pSmad1/5, p-p38, and pERK, but the presence of pJNK in the E14.5 control condyle (A, C, E, G), and the presence of pSmad1/5, but absent pJNK as well as p-p38 and pERK in the transgenic condylar cartilage (B, D, F, H). Abbreviation: C, condylar cartilage; G, glenoid fossa; M, Meckel's cartilage. Scale bar = 100 µm.

We have shown previously that the expression of *caBmprIa* in CNC lineage induces ectopic activation of Smad1/5/8 signaling as well as p38 signaling in the developing palatal shelves [51]. We therefore set to examine alterations in BMP canonical and non-canonical signaling pathways in the condylar cartilage of *Wnt1-Cre;pMes-caBmprIa* mice by immunohistochemistry. Interestingly, we detected no activation of Smad-dependent as well as p38 and Erk1/2 pathways in the control condyle, as assessed by the lack of pSmad1/5, p-p38, and p-Erk1/2, but observed activity of p-JNK signaling (Fig. 7A, 7C,7E, 7G). In contrast, the condylar cartilage of *Wnt1-Cre;pMes-caBmprIa* mice exhibited an ectopic activation of pSmad1/5, but an absence of pJNK signaling, along with unaltered p38 and pEek1/2 pathways (Fig. 7B, 7D; 7F, 7H). These observations indicate that the switch between BMP canonical and non-canonical signaling pathways likely underlies the fate conversion from the secondary to primary cartilage, with the Smad-dependent signaling favoring the primary cartilage fate.

## Discussion

Compared to synovial joint formation in the appendicular skeletons, TMJ development and the underlying molecular mechanisms are relatively under-studied. While the critical roles of BMP signaling in long bone joint development and homeostasis have been well documented [21], [26], [29], its role in TMJ formation remained completely unknown. In this study, we present evidence that BMPRIA mediated signaling is essential for TMJ morphogenesis, and overly activated BMPRIA signaling is detrimental to TMJ formation. Our results also reveal apoptosis in the interzone as a potential cellular mechanism for cavitation of the TMJ, similar to that in long bone joint formation.

### A BMPRIA-Ihh positive regulatory pathway regulates TMJ development

It has been well established that BMP and Ihh signaling interact to regulate chondrocyte proliferation and hypertrophic differentiation [34], [53]–[55]. In the developing limb, BMP signaling, particularly the BMPRIA mediated pathway, positively regulates *Ihh* expression that could also activate in the perichondrium the expression of several BMP ligands, forming a BMP-Ihh positive feedback loop [34], [53], [54]. Although there is no evidence for an interaction of BMP and Ihh signaling in joint development, the fact that several BMP ligands and receptor are expressed in the interzone and that mutations in either *Noggin* or *Ihh* lead to joint defects including joint ablation in limbs implies the existence of such interaction [15], [21], [28]. Indeed, in the current study, we found that the ablation of *BmprIa* in CNC lineage produces TMJ defects resembling that in *Ihh* mutant. In both mutants, a functional TMJ failed to form, evidenced by the absent Lubricin expression (Fig. 2) [16]. In addition, both mutants displayed a lack of a distinct articular disc due to failed disc separation from the condyle, persistence of the interzone cells, as well as absent synovial membrane on the articular surface of the glenoid fossa. Consistent with Ihh function in disc formation and separation during TMJ morphogenesis [16], [17], we found a dramatic down-regulation of *Ihh* expression in the developing condyle of *Wnt1-Cre;BmprIa^f/f^* mice. This result also indicates that similar to its role in developing appendicular skeletons, BMPRIA mediated signaling also acts as a positive regulator of *Ihh* expression in the condylar cartilage. On the other hand, *BmprIa* expression was significantly reduced in the condyle and interzone of the *Ihh* ^-/-^ TMJ, suggesting the existence of a BMPRIA-Ihh positive feedback loop in the developing TMJ. However, the delayed hypertrophic differentiation observed in the condylar cartilage of *Wnt1-Cre;BmprIa^f/f^* mice appears to be opposite to the premature hypertrophic differentiation defect seen in the *Ihh* ^-/-^ condyle as well as in the long bones of mice carrying *BmprIa* deletion in chondrocyte lineage [16], [34]. Although the underlying mechanism is currently unknown, the discrepancy would likely be attributed to the residual *Ihh* expression in the *Wnt1-Cre;BmprIa^f/f^* condyle as well as the condyle's property as secondary cartilage.

**Figure 8:**
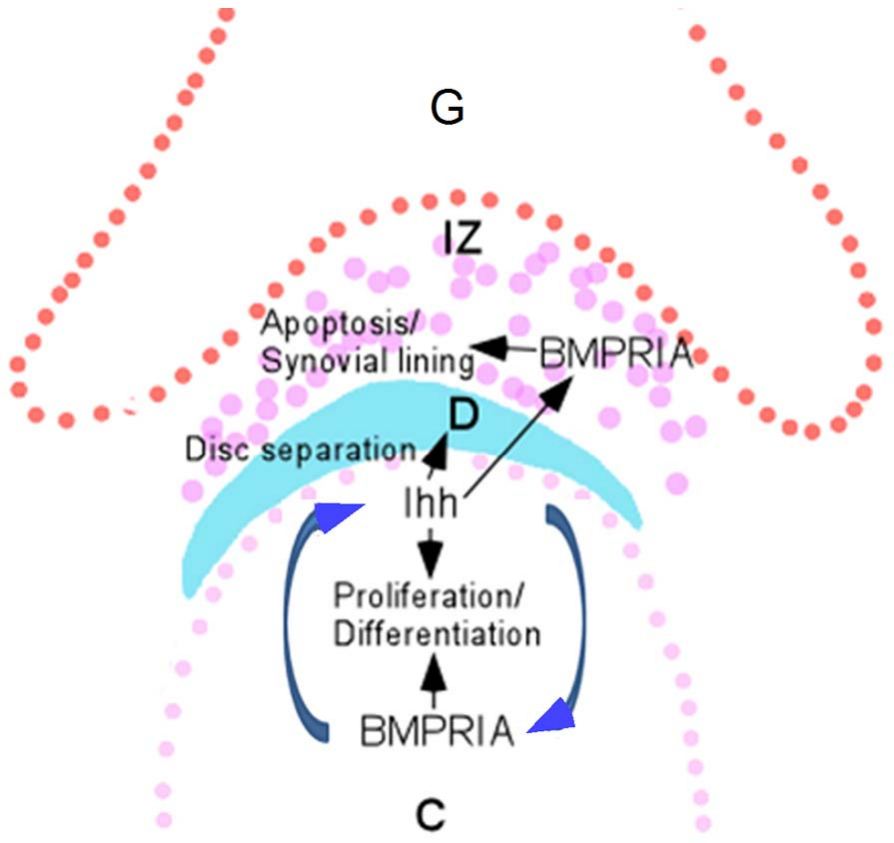
A model illustrating the interaction between BMPRIA and Ihh and their functions in regulating distinct steps during TMJ morphogenesis. Abbreviation: C, condyle; D, disc; G, glenoid fossa; IZ, interzone.

Nevertheless, based on above mentioned observations and the established roles for BMP and Ihh signaling in limb development, we propose a model to summarize the function of the BMPRIA-Ihh regulatory pathway in regulating distinct steps during TMJ morphogenesis (Fig. 8). In this model, BMPRIA and Ihh regulate the expression of each other to coordinate chondrocyte proliferation and differentiation in the developing condyle. Meanwhile, Ihh, produced in the condyle, diffuses into the interzone to regulate disc separation and to maintain the expression of BMPRIA that in turn acts in a cell autonomous manner to regulate synovial membrane formation and to trigger apoptosis.

### Augmented BMPRIA signaling in CNCs converts the secondary cartilage of the condylar primordium to primary cartilage

Despite being a secondary cartilage, the condyle shares many similarities with primary cartilage in development, including the expression of genes known to be important for cartilage growth and differentiation such as *Bmp2*, *Bmp7*, *Sox9*, *Runx2*, *Osterix*, *Ihh*, *Pthrp*, *Vegf*, *Col II* and *Col X* [8], [16], [17], [35], [56], [57]. However, the condyle also differs from primary cartilage by its expression of Col I and Col II simultaneously and its capability of differentiating into either chondrocytes or osteoblasts [52]. Although the underlying mechanism for fate determination of primary v.s. secondary cartilage remains elusive, our results that augmented BMPRIA signaling is able to convert the condylar cartilage to a primary cartilage, evidenced by the lack of *Col I* expression, implicate BMP signaling in such fate decision. *BmprIa* is expressed in the cartilage condensations of both long bone and condyle (this study) [33], suggesting its role in the fate decision of both primary and secondary cartilages. It appears that a tightly tuned BMPRIA signaling is essential for fate determination of secondary cartilage. Accompanied with this fate conversion is the switch of the downstream BMP signaling pathway from the non-canonical JNK signaling to the Smad-dependent pathway in the condylar cartilage, suggesting that higher activity of BMPRIA signaling preferentially activates the Smad-dependent pathway, which favors primary cartilage formation. Indeed, the lack of Smad-dependent signaling in the condyle (this study) and the strong expression of pSmad1/5/8 in the limb cartilage condensation [33] further support this notion. Furthermore, consistent with the role of BMPRIA signaling in apoptosis in the developing limb [26], [32], overly activated BMPRIA signaling causes extensive apoptosis in the condylar cartilage and leads to condylar cartilage degeneration, indicating a detrimental effect on chondrocyte survival.

Despite an essential role for *BmprIa* in chondrogenesis and endochondral bone formation, in our current study, we found that inactivation of *BmprIa* did not affect glenoid fossa osteogenesis, suggesting that *BmprIa* may not be essential for intramembranous bone formation. However, elevated BMPRIA signaling instead inhibits osteogenesis in the glenoid fossa. Thus, although BMP signaling is generally accepted as a positive regulator of osteogenesis, elevated BMP signaling could have an opposite effect, depending on the tissue and cell types. Since a normal developing condyle is required to sustain the development of the glenoid fossa [9], the degeneration of the glenoid fossa in *Wnt1Cre;pMes-caBmprIa* mice could be the consequence of failed osteogenesis, or result from an abnormal condylar cartilage with altered property, or both.

### Apoptosis as a cellular mechanism of TMJ cavitation

Cell death in the middle of the interzone is considered the cellular mechanism for physical separation of the contiguous cartilage elements during joint formation in long bones [21], [58]–[61]. In the developing TMJ, although the primordial condyle and glenoid fossa form independently and become approximately through condylar growth, disappearance of the interzone cells is necessary for the formation of a joint cavity. The interzone mesenchymal cells are believed to contribute to the articular disc, capsule, and the synovial membrane of the joint cavity [3], [4]. However, if apoptosis occurs in the interzone of the TMJ remains arguably. It was reported previously that in the rat TMJ at late developmental stage, apoptotic cells were found only at the subsurface of the condyle and in the region at which the lateral pterygoid muscle attached to the condyle, suggesting that apoptosis may be associated with the lower joint cavity formation of the TMJ [62]. However, in our studies, we found extensive cell death in the interzone of the control TMJ at E15.5, right before the upper cavity becomes discernibly at E16.5. However, in the *Wnt1-Cre;BmprIa^f/f^* TMJ, such extensive apoptosis was not observed, consistent with the pro-apoptotic role of BMPRIA mediated signaling. The discrepancy between our results and that by Matsuda and colleagues [62] could be attributed to the stage difference. Nevertheless, the lack of apoptosis appears to contribute to the persistence of interzone cells in the mutant TMJ. In addition, BMPRIA signaling is also required for organization of some interzone cells to become synovial lining layer, which could also contribute to the cavitation of the TMJ. We thus propose that the upper joint cavity of the TMJ is formed by organization of the interzone cells into synovial lining layer and capsule, and by removal of excessive cells via apoptosis.

In conclusion, our studies using transgenic loss-of- and gain-of-function approaches reveal the importance of BMPRIA mediated signaling in TMJ morphogenesis and establish a BMPRIA-Ihh positive regulatory pathway in controlling disc separation, synovial membrane formation, as well as joint cavity formation.
